# An empirical study of feature extraction for Hubei folk art of carved paper-cutting

**DOI:** 10.1371/journal.pone.0311923

**Published:** 2024-10-23

**Authors:** Zhen Yang, Yanqi Liu, Weiling Wang, Yu Li, Xiuxia Yuan

**Affiliations:** 1 Xuchang Innovation Center of Intelligent Construction and Building Industrialization Technology, Zhongyuan Institute of Science and Technology, Zhengzhou, Henan, China; 2 School of Art Design, North China University of Water Resources and Electric Power, Zhengzhou, Henan, China; 3 Henan North China Water Resources and Electric Power Survey and Design Co. Ltd, Zhengzhou, Henan, China; Nanchang University, CHINA

## Abstract

Excellent traditional culture is the root and soul of a nation, which is the carrier of historical precipitation and continuous development. In this aspect, Hubei folk art of carved paper-cutting has important cultural value and historical significance in the composition of Chinese traditional culture, which is a national intangible cultural heritage under key protection and urgently needs to be carried forward. This paper carried out an empirical study using the survey and comparative analysis methods on the feature extraction for Hubei folk art of carved paper-cutting. Through investigation and comparative analysis of vast artworks of Hubei carved paper-cutting, the classics are selected as the representative to extract the regional characteristics, the artistic values, and the composition and modelling methods. Results indicate that the common features exist on the artistic values in beautification of life and education, the composition principles of central-based, symmetry, balanced or conformal, and the modelling techniques using Yin and Yang carving, unobstructive perspective, distortion and exaggeration. However, the regional features in cultural expression, folklore communication, modelling skills are apparent and strong interlinked with the regional historical development in social activities, labor productivity and surrounding environment. This study provides a multi-dimensional perspective for the modern inheritance and development of traditional Hubei artworks of carved paper-cutting with specified characteristics.

## 1. Introduction

Paper-cutting as a style of art exists in dozens of countries such as China, Japan, Korea, India, Indonesia, Thailand, Myanmar, Vietnam, Mongolia, Australia, Russia, Poland, Denmark, Switzerland, Netherland, French, Germany, UK, USA, Egypt, Greece, Turkey, etc. Nowadays, artworks of paper-cutting spread in countries around the world, promote cultural exchanges, and become the artistic bridge of spreading friendship [1]. Specially, paper-cutting is an important part of many traditional folk arts in China. Before the invention of paper, paper-cutting type engrave art has appeared in ancient times of the Chunqiu Warring Period from 770 to 221 BC, when people use cloth, leather, gold leaf and other thin materials to create exquisite patterns through cutting, carving, engraving and other technics [1–3]. To the Western Han Dynasty in 105 DC, paper began mass production with innovated paper technology, which promotes engraving techniques gradually evolved into paper-cutting art forms. Because of the prevailing of witchcraft culture in ancient Jingchu (short name of Hubei region), paper-cutting spread widely in its area [1, 4]. During the Southern and Northern Dynasties from 386 to 581 DC, it was spread to be used as a decoration for festivals and customs, meaning of warding off evil and seeking good luck [5, 6]. In Tang Dynasty period from 618 to 907 DC, paper-cutting art began to integrate into social folklore, craft production, decoration and beautification and other aspects of life, its numerous themes, rich content, skilled technique make people breath-taking [4, 6]. In the Song Dynasty from 960 to 1279 DC, paper-cutting technology became more mature and sophisticated, and many types of paper were available to make paper-cutting works. The prospered folk commercial culture excited the application of folk paper-cutting, there are specialized in paper-cutting artists. In addition to folk sacrificial paper cutting, there are fireworks, window flowers, lantern decoration and others [7–9]. With the rapid development of commodity economy in the Ming and Qing dynasties (1368–1912 DC), the traditional handicraft industry flourished, and the level of paper-cutting art was also significantly improved. Paper-cutting art became increasingly specialized and commercialized, and artisans created more exquisite handicrafts. This was also a stage for Hubei paper-cutting art to mature and prosper. Paper cutting was not only used for decoration and festival, but also for embroidery patterns of flowers in articles of shoes, hat, bib, pillow and so on. With the increase in market demand, in order to improve production efficiency, paper-cutting technic was transformed from the original scissors to use a small carving knife. This is the formation of Hubei folk art of carved paper-cutting [9, 10].

In Minguo period from 1912 to 1949 DC, due to social unrest and locating in the middle and lower reaches of the Yangtze River, Hubei always faced floods that occurred year after year in a long and concentrated rainy season, making the life of common people extremely difficult. This compels people to learn the carved paper-cutting techniques to make a living. In this aspect, male artists have physical advantages in the convenient and safe travel around, the creation subject of Hubei carved paper-cutting was gradually changed from peasant women in the early days to male professional artists [9–11].

With the establishment of PR China, Chinese people coming into a new society, Hubei folk art of carved paper-cutting develops with new vigor and vitality. Breaking through the tradition and innovating bravely, Hubei folk art of carved paper-cutting is no longer limited to traditional themes. A large number of carved paper-cutting works have been created to praise the new era and new life. The content of the works is much closer to daily life, era development or political current affairs, coming into Chinese family life and publicity medias such as newspapers, magazines, posters, cultural presentation board, and electronic media [1, 3, 11, 12].

Therefore, as an artistic form, Hubei folk carved paper-cutting deeply rooted in the primitive emotions and life instincts of the Chinese nation, bearing the psychological imprint of the evolution of national culture for thousands of years. It not only directly presents the most primitive and simple cognitive thinking about the relationship between nature and society, but also integrates the unique artistic visual thinking mode. This kind of modelling concept is simple and natural, which is the free display of the spirit and behavior of the creative subject, and is also the unique charm of carved paper-cutting. Hubei carved paper-cutting works, represented by those crated in Xiaogan, E’zhou and Xiantao cities, are delicate and beautiful, finer in a subtle way, harmony between virtual and real, fluent in knife technique, and neat with complexity, with rich themes and forms and high artistic appreciation value [13–16]. They are living fossils with distinct regional characteristics and profound cultural deposits that reflect the production and life of people in Hubei and convey their emotions. In June 2008, Hubei folk art of carved paper-cutting was selected into the second batch of National Intangible Cultural Heritage Expansion Projects List [2, 12, 15].

Nowadays, with the continuous integration of economic globalization and modern multi-culture, the relationship between traditional culture and modern design has become a frequent topic in the field of art design [2, 11, 12]. Although having a long history and profound cultural heritage, Hubei carved paper-cutting still needs to carry forward the excellent traditional culture on the basis of reform and innovation in line with the social development [12, 15, 17]. This is both a requirement for self-improvement and a need for reaching new height of art design to express people’s aesthetic consciousness and cultural pursuit. Therefore, deep excavating the relationship between carved paper-cutting and modern design, and exploring the feasibility of their combination can enrich the theoretical research of carved paper-cutting art, and also expand the application form of carved paper-cutting using the current design techniques and manufacturing technology. This highlights the significance of increase the cultural value to modern design works, helping promote the organic combination of the innovative development of excellent traditional art with the constructing a modern sustainable society.

Moreover, current researches have been concentrated on the origin and development history, the culture and folklore expression, the manufacture techniques, the inheritance and innovation methods, and the protection strategy of Hubei folk art of carved paper-cutting. However, in a long history of Hubei carved paper-cutting, the characteristics of artworks are mainly inherited and carried forward by the folk artists. Although the Hubei folk art of carved paper-cutting was selected into the second batch of National Intangible Cultural Heritage Expansion Projects List in June 2008, it still faces an impact of rapid development of economic society and cultural fissions. This is urgent of need to do some works to carry forward the excellent traditional art, especially a study of feature extraction that could be learnt, generated and designed by computers [18–20]. This can promote a mass design and production under abundant needs in many situations such as traditional festival or several kinds of celebration ceremony. Therefore, an empirical method was applied to focus on the features extraction for Hubei carved paper-cutting. The comparative analysis was performed for vast artworks saved in Xiaogan Carved Paper-cutting Heritage Center, Xiantao Mass Art Museum, Ezhou Huarong Intangible Cultural Heritage Exhibition Hall, and Wuhan Mass Art Museum, and those downloaded on websites and published in literatures. The authors of this paper visited the representative inheritors and directors listed in the Acknowledgement part. Based on the results of empirical activities, this paper selects classic artworks for in-depth analysis to help a better understand the artistic characteristics of Hubei carved paper-cutting. Three aspects are summarized for Hubei carved paper-cutting on regional features, artistic values, and composition and modelling methods. The results are valuable for artists in creation process and viewers in participation to master the essential meanings and produce an emotional resonance from the artworks of Hubei carved paper-cutting.

## 2. Regional features of Hubei carved paper-cutting

Hubei province is located in the central part of China, which has a variety of landforms, surrounded by mountains on three sides and low in the middle. In addition to the Yangtze River and Han River, thousands of rivers and lakes are in the region. Therefore, she is known as the "province of thousands of lakes" with rich water resources. Influenced by geographical location and folk customs, different regions have different regional culture. This gives regional features of the carved paper-cutting, reflecting their own cultural background and aesthetic customs. In this section, the most representative carved paper-cutting of Xiaogan, E’zhou and Xiantao are taken as the main objects to analyze the unique regional history and artistic charms.

### 2.1 The filial piety culture of Xiaogan City

Xiaogan City, located in the northeast of Hubei province, China, has a profound filial piety culture since ancient times. In the year of 453 AD of the Southern Dynasty, the filial piety was vigorously promoted. In the year of 454 AD, a county was set up and given the name Xiaochang which means the prosperity of filial piety in a local folk style. In 924 AD, the name of Xiaochang was changed to Xiaogan according to the folk stories moved gods about three filial sons of Yong Dong selling his body to bury his father, Zong Meng crying bamboo shoots, and Xiang Huang warming bedclothes and fanning pillow. This always reminds people here to worship filial piety and perform filial piety [13]. With a long historical development, filial piety forms the basic culture of the traditional culture of Chinese nation. Therefore, the flourishing carved paper-cutting in Xiaogan City creates a broad mass base, which is an important mode of transmission and material carrier of the filial piety culture marked by regional characteristics.

**Fig 1:**
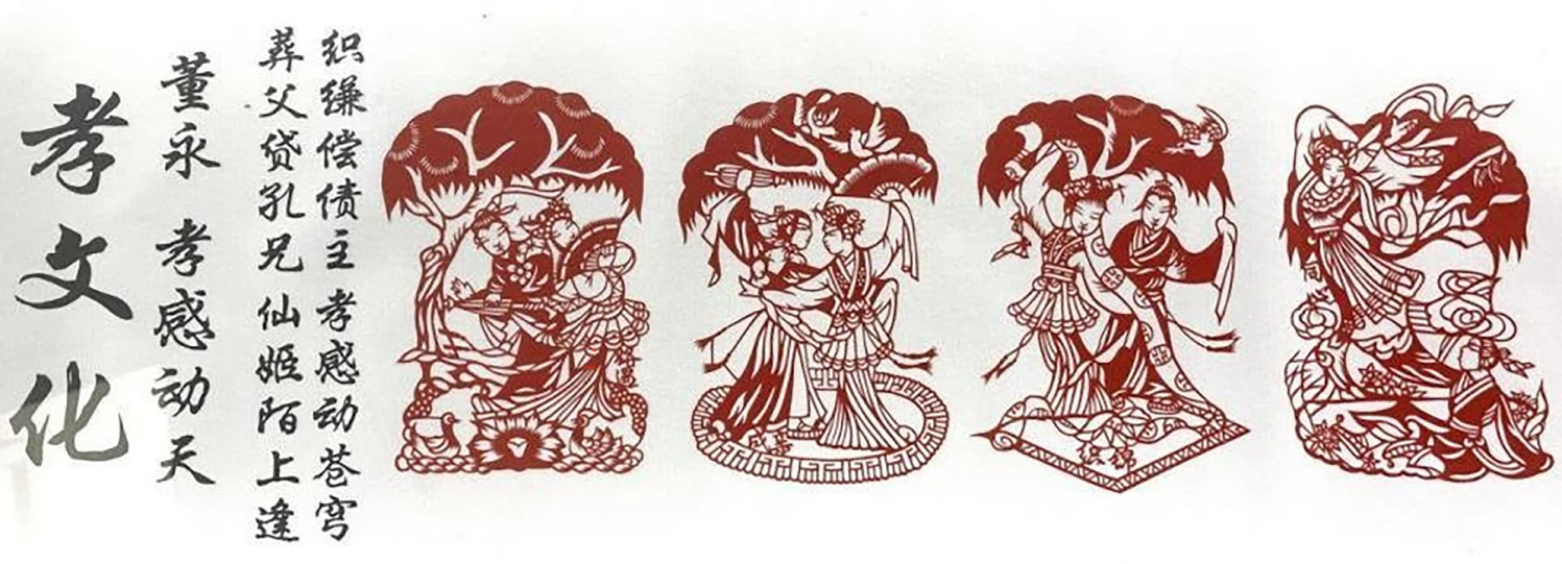
”Shadow of a Pagoda Tree” created by Z. Zhong and F. Chi.

Fig 1 is a picture of the classic theme of carved paper-cutting artwork saved in Xiaogan Carved Paper-cutting Heritage Center, Xiaogan City, China, which can be downloaded online: https://baijiahao.baidu.com/s?id=1605380213236875183. It tells a folk story about a filial son named Yong Dong according to the version of Yong Dong and Zhinu [17, 21]. The artwork named as Shadow of a Pagoda Tree is a combined composition with four small stories for creation, each of which is a beautiful work even if viewed individually, and when combined together, it becomes a complete, highly graphic and story-oriented carved paper-cutting work. The Chinese words on the left of picture are in title of Filial Culture, and Yong Dong’s filial piety touching Gods, while the four poetic lines tell the main contents of the story. The four pictures focus on the second half of the story, which depicts Yong Dong, who sells himself into slavery and works hard to bury his father, meeting a fairy who is moved by his filial piety under a pagoda tree. They fell in love and wedded, and supported each other after marriage; after helping Yong Dong repay his debt through weaving cloth, the fairy told him who she is, and flied away to the heaven. This expresses that just because of Yong Dong in different periods with moral characters of respect, filial duty, good faith and know how to be grateful, the fairy was moved to help him. The vivid artwork is a refined expression of the four characters of love, respect, sincerity and kindness in Chinese humanist spirits.

### 2.2 The historic background of E’zhou City

E’zhou City is located in the southeast of Hubei, on the south bank of the middle reaches of the Yangtze River. E’zhou has a long history, splendid culture, rich natural resources and easy to cultivate. Since ancient times, agricultural civilization has been developed and became an important origination of Jingchu culture in eastern Hubei. With crisscross lakes, whenever the rising season, people have to leave their homes for survival, wandering around, rely on busking to make a living. Because most of the carved paper-cutting artists in E’zhou grew up in the villages along rivers and lakes, fish is a naturally frequent visitor in paper-cutting works [14]. E’zhou City is the hometown of Wuchang fish. Since ancient times, this fish has been intoned with a special meaning by many literati and writers, and many cultural phenomena such as Wuchang fish poetry culture and Wuchang fish food culture have become important themes of E’zhou carved paper-cutting art [22].

**Fig 2:**
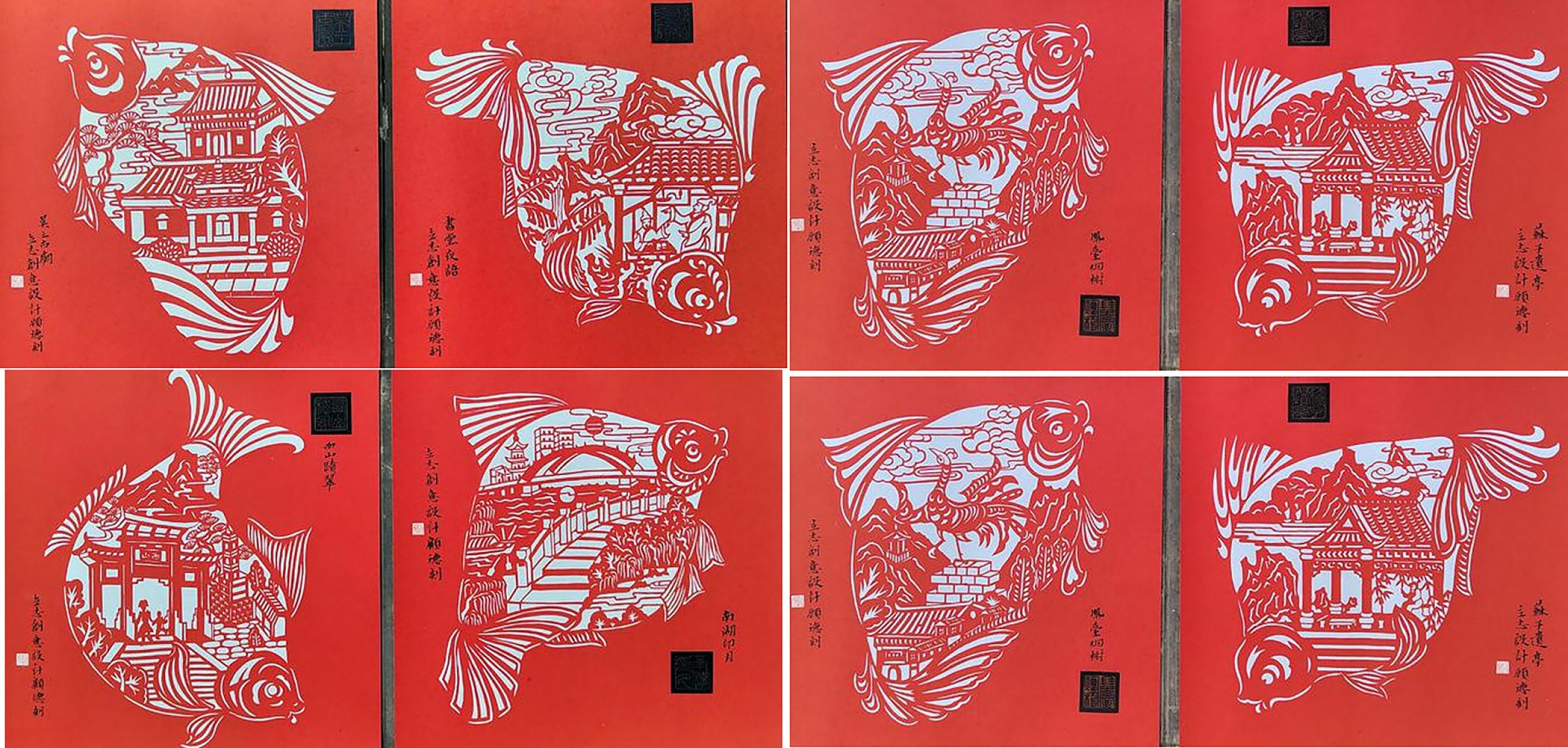
“Wuchang fish and E’zhou ancient Eight Scenes” created by L. Li and D. Gu.

Fig 2 is the picture showing the artworks of carved paper-cutting which perfectly composite Wuchang fish with representative ancient Eight Scenes of E’zhou, which can be downloaded online: http://www.ezhou.gov.cn/. Differed from the past E’zhou carved paper-cutting of independent Wuchang fish and Eight Scenes, this set of works uses the graphic design method of displacement isomorphism, the ancient Eight Scenes organically integrated into the body of Wuchang fish, each picture is extremely vivid and readable. Eight shapes of Wuchang fish are different and beautiful, not only have no repeat shape of fish head, fin and tail, the decoration is also unique to be crescent, chrysanthemum, peony or organized using long and short lines and curves. The picture is very clever, the fish seems to be swimming in the water Eight Scenes, but also like playing with companions. Wuchang fish here is not only as an animal pattern, but also the design technic gives it a humanistic sense, perfectly reflecting the regional cultural characteristics of E’zhou and Wuchang fish culture.

### 2.3 The water village style of Xiantao City

Xiantao City is located at the junction of the Yangtze River and the Han River. It is known as the "hometown of fish and rice". where the folk customs are simple and honest, the waterways are connected, and the products are abundant. Carved paper-cutting is quite representative among folk arts [15]. The geographical location with rivers and lakes nourishes this fertile land of Xiantao, but brings frequent flood disasters in frequency that is at the top of the list in Hubei before the foundation of PR China. Because the natural disasters took place in uncertain years, people here were engaged in various crafts to support their families. With a low cost and a large demand, carved paper-cutting became a common craft, which is also created with the representative elements of water village such as lotus, lotus root, fish and shrimp, under the impact of its special geographical factors and living environment [23].

**Fig 3:**
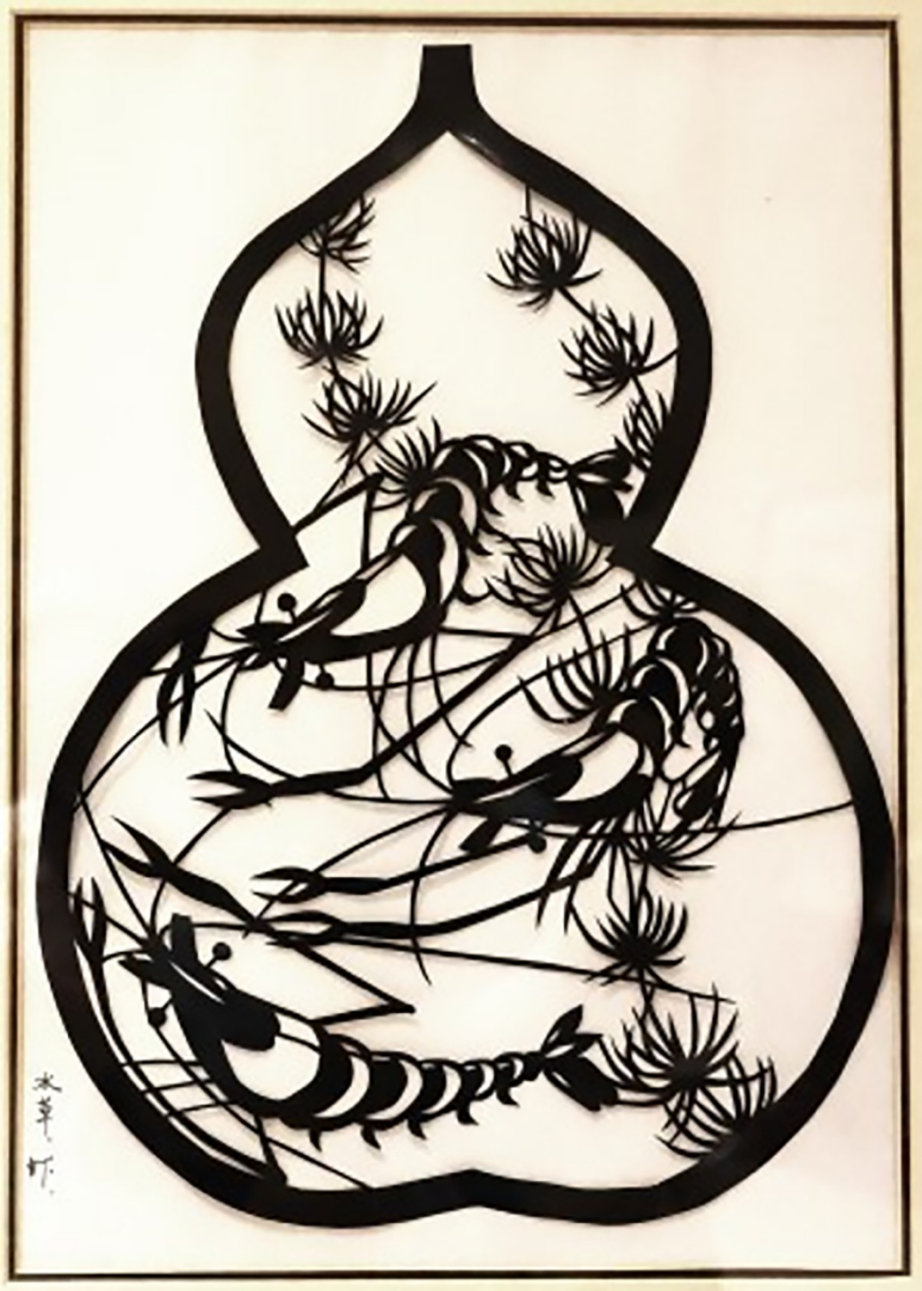
Artwork of "water grasses and shrimps".

As the artwork saved in Xiantao Mass Art Museum, Hubei, shown in Fig 3, inside the outer frame in the shape of a gourd, the scene of shrimp playing among the water grasses is vividly depicted. The local water town elements of gourd, water grass and shrimp are cleverly integrated into the symbolic form space of gourd, meaning the beauty and richness of life.

## 3. The artistic values of Hubei carved paper-cutting

### 3.1 Beautification of life

**Fig 4:**
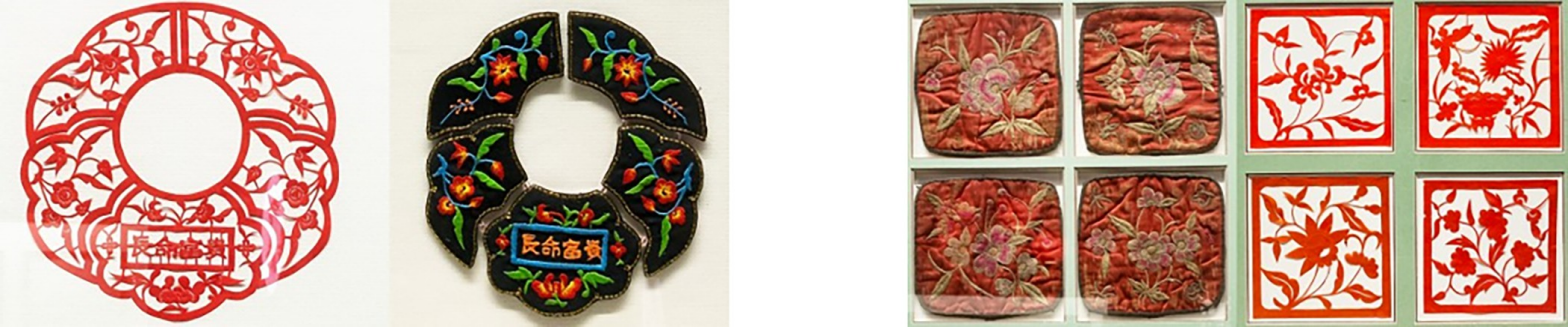
Artworks of carved paper-cutting. (a) Children’s saliva pocket, (b) Pillow flowers.

Hubei carved paper-cutting is deeply rooted in People’s daily life, which is not only used for etiquette and festival decoration, but also used as an embroidery base. Therefore, carved paper-cutting and embroidery are closely linked "sister art", male paper-cutting skills and female embroidery skills complement each other, the creation mode of "male engraving and female embroidery" is the unique feature of Hubei carved paper-cutting. The women bought the auspiciously meaningful paper-cutting patterns from the paper-cutting artists, and embroidered them on various items to pray for a happy life. For instances, the clothing type of hat flower, shoe flower, apron flower, saliva pocket flower and shoulder flower, the decorative home type of tent edge flower, pillow flower, quilt flower [24]. These categories are divided into a variety of styles and uses. Fig 4 shows the artworks saved in Xiaogan Carved Paper-cutting Heritage Center, Xiaogan City, China. The meaning of Chinese words on Children’s saliva pocket is a long life of abundance and respectability. The pillow flowers are often embroidered on the ends of a pillow, which are composited with auspiciously meaningful flowers or plants. Nowadays, the traditional paper-cutting works that have survived are mainly based on conformal dress patterns, plants and flowers are mostly in the pattern, while dragon, phoenix, bird and fish are also the most in animal theme. The carved paper-cuttings is not only a skill to beautify life, but also an important medium to express inner feelings and wishes, which present exquisite appearance on ordinary clothing and household items to give a unique color and beauty to file people’s heart with pleasure and satisfaction.

**Fig 5:**
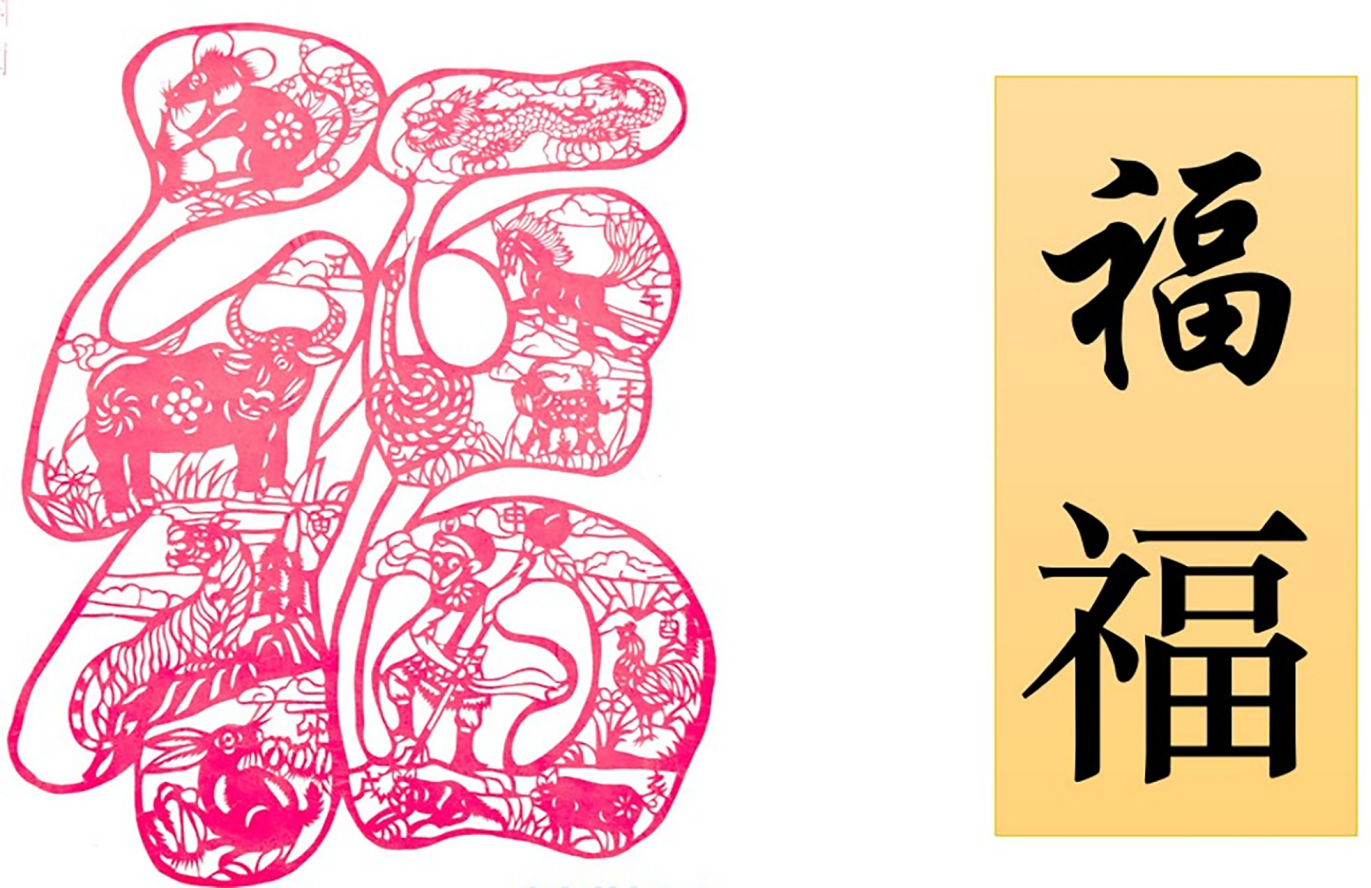
Artworks for home decoration created by F. Chi. (**a**) Artwork, (**b**) Chinese word “Fu”.

As a kind of ceremonial activity, the birthday ceremony is usually a way for the younger generation to express respect and blessing to the elders or relatives and friends. On the occasion of celebrating the birthday, the carved paper-cutting often takes the elements of longevity such as the peach, the longevity god, and the crane as the theme, which are rich in symbolic significance, and convey the deep blessing and high respect. As presented in Fig 5 that can be downloaded online: https://hb.cri.cn/2019-02-02/5216d6d4-56c2-61c6-4f71-342932e55725.html, a Chinese word “Fu” leaps into view vividly, which is intertwined together, both in shape and spirit. Moreover, the twelve signs of Chinese Zodiac accompanied with the earth branch in Chinese Gan-Zhi’s dating method are also presented in lifelike, that is in sequence of mouse, cow, tiger, rabbit, dragon, snake, horse, goat, monkey, chicken, dog and pig. The twelve Chinese words marking the earth branch in Chinese Gan-Zhi’s dating method are skillfully arranged in the corresponding animal signs to play a role of linkage and beautify the whole figure. The ingenious layout and caving intricacy of this artwork are classics in Hubei folk art of carved paper-cutting.

### 3.2 Folklore communication

At the beginning of its birth, the carved paper-cutting is close to the material production and human spiritual production. This is consistent to the intertwin of the artistic creation with the witchcraft and religious activities in primitive society. Historical records show that the paper-cutting originated from the early witchcraft sacrificial function, and the purpose of witchcraft is to eliminate disease to disaster, exorcise ghosts, divination luck and doom. This gives the carved paper-cutting a function of seeking blessings. With the social development, the witchcraft gradually disappeared and evolves to be a folk cultural activity. Therefore, the carved paper-cutting still plays an indispensable role in traditional annual festivals, weddings and funerals or other folk activities.

#### 3.2.1 Traditional festival folk custom

Putting up door paper-cuttings during the Spring Festival is a traditional custom in Hubei, China. People believe that door paper-cuttings can bring good luck and auspice. A set of door paper-cuttings is five pieces, pasted on the lintel as decoration, and posted together with Spring Couplets on the 30th of the Chinese New Year to welcome the arrival of the New Spring. The color of the door paper-cuttings will be different according to the purpose: red for festivals, and white during the mourning period, which cannot be confused. With different shapes and rich patterns of the Chinese words Fu, Shou and Shuangxi representing fortune, longevity and happiness, and the flowers, characters or animals, they are rich in profound cultural connotations and auspicious meanings. When the spring breeze blows, the door paper-cuttings will flutter with the wind like flags, adding a strong festive atmosphere to the festival.

**Fig 6:**
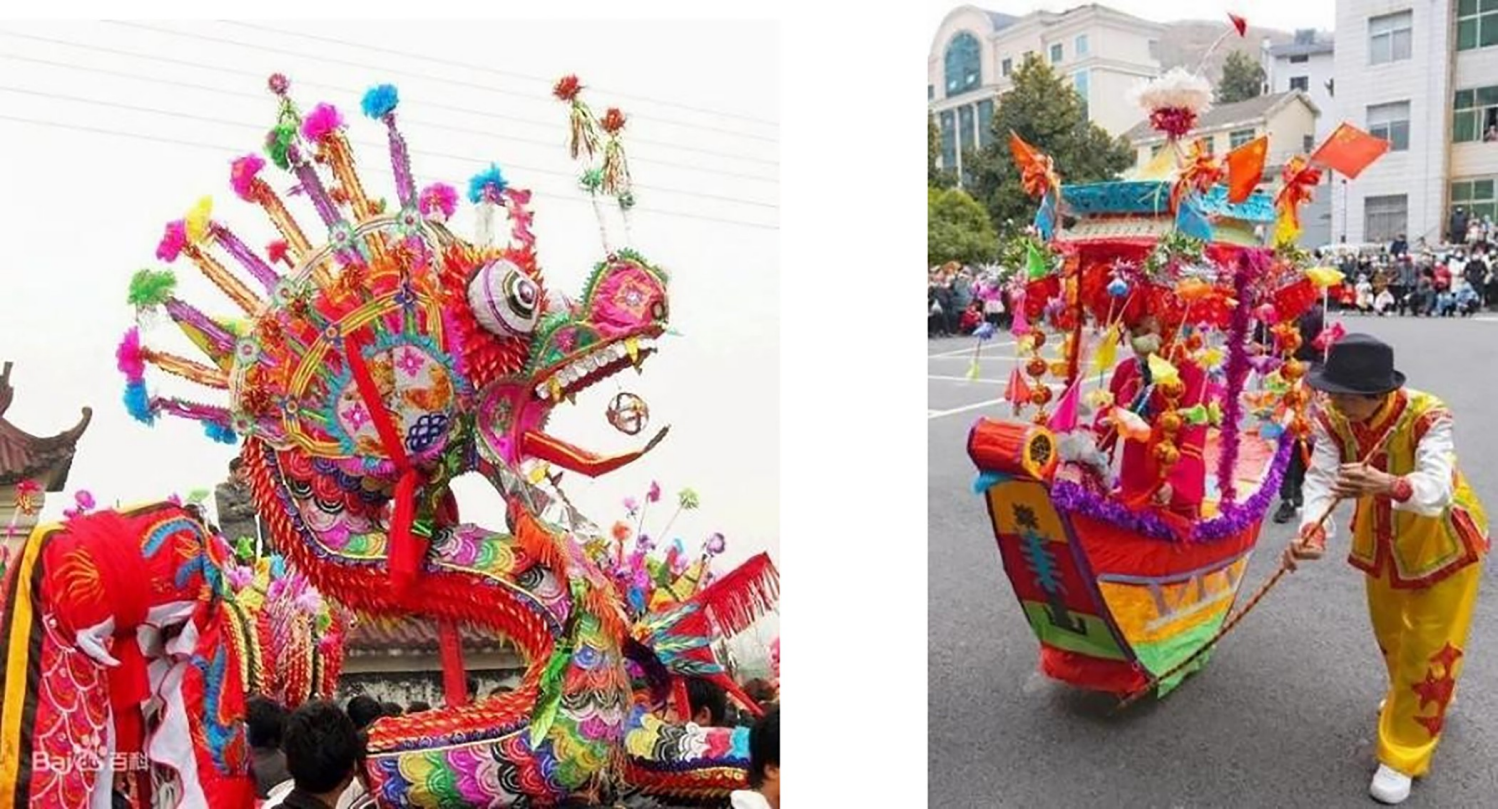
Artworks of carved paper-cutting. (**a**) High Dragon, (**b**) Lotus gathering boat.

The carved paper-cuttings can also be seen everywhere in the Lantern Festival. Among them, the dragon lantern and lotus boat are the most representative. Fig 6 exhibits the artworks that combine the folk art of carved paper-cutting with bamboo tying, which can be downloaded online: https://image.baidu.com/. High dragon dance, lion dance and rowing lotus boat are essential annual custom activities in the Spring Festival in Wuhan, Xiaogan, Xiantao and other places of Hubei, China, in order to pray for good weather and grain harvest. The dragon head and tail are in the S-shape with bamboo strips and colorful paper, while the carved paper-cuttings with specific patterns are affixed to the corresponding position. The lotus boat is a kind of traditional folk song and dance form evolved from the scene of collecting lotus by boat. The top of the boat is decorated with colorful paper-cuttings and flowers, and its production method is similar to the high dragon.

**Fig 7:**
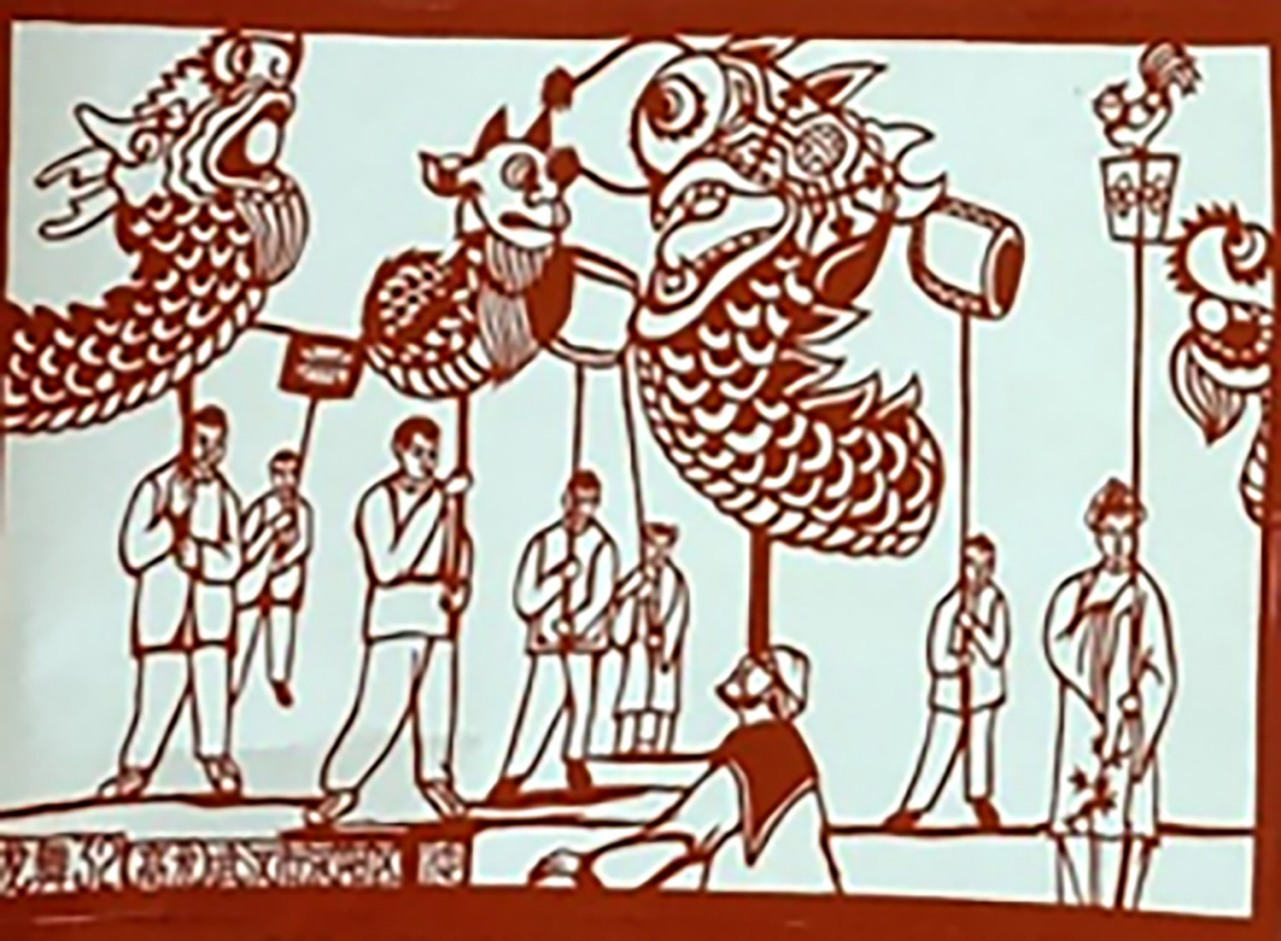
Artworks “Dragon dance” created by F. Chi.

Additionally, the carved paper-cutting achieves an effective dissemination of festival culture, which provides daily feelings of the charm of festival culture with the appreciation of paper-cutting artworks with depict various festival scenes and traditional customs. As the pictures of Fig 7 for the artworks of “Dragon dance” saved in Xiaogan Carved Paper-cutting Heritage Center, Xiaogan City, China. It is a scene of a dragon dance performance during the Spring Festival in Hanyang District, Wuhan, people use bamboo poles to pick up the dragons and let them make beautiful jumps and swings in a cheerful atmosphere, expressing happy mood of people to welcome the Chinese New Year.

#### 3.2.2 Life etiquette

Life etiquette is a person in different life stages or occasions, follow a certain code of conduct and social norms to express respect, care of social behavior. It covers every important moment and event throughout a person’s life, such as birth ceremony, first birthday ceremony, wedding, birthday ceremony and funeral. Nowadays, the carved paper-cutting is widely used in the main events of wedding, birthday celebration and funeral ceremony.

**Fig 8:**
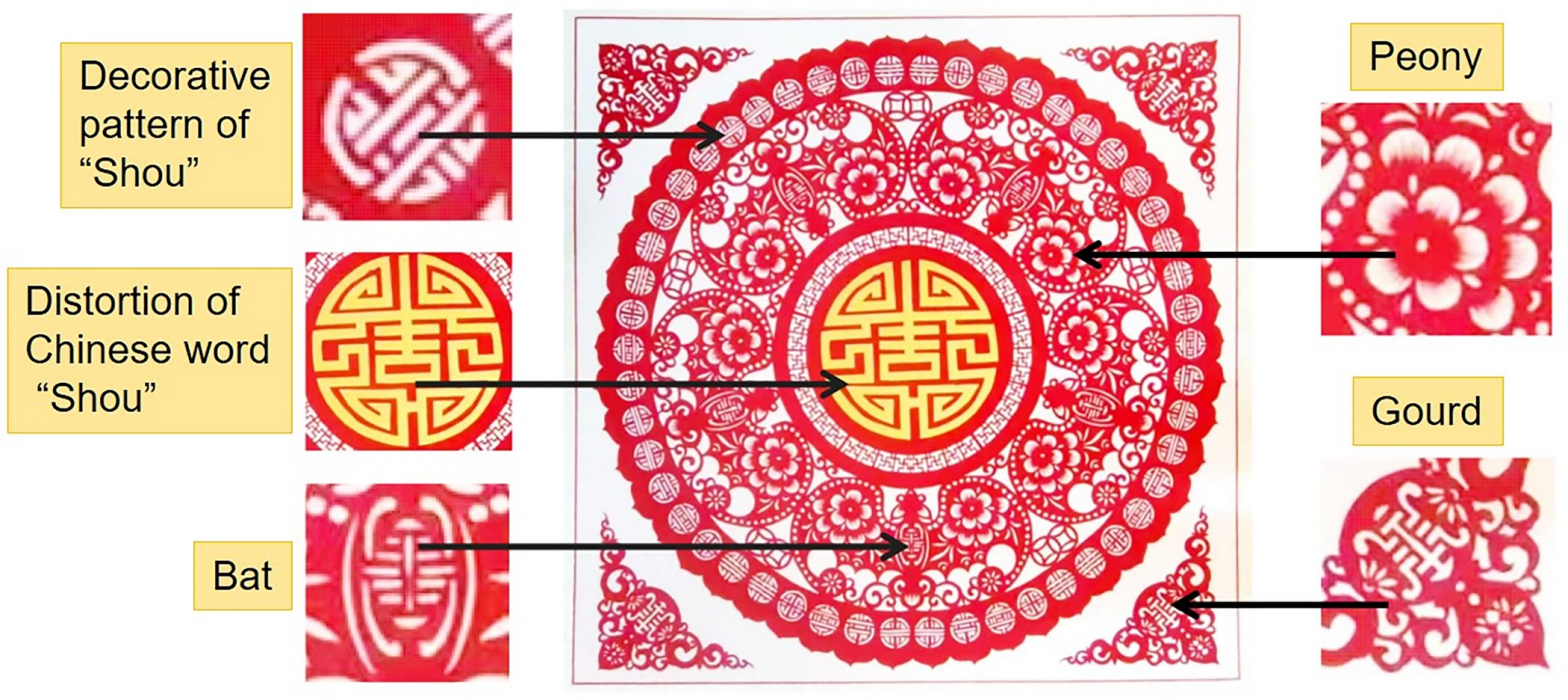
Artworks “Five Fu hold Shou” for birthday ceremony created by Z. Xia.

In the wedding custom, carved paper-cuttings are known as "happy flowers" which are mostly used to decorate betrothal gifts, bedding, clothing boxes, windows and doors of dressing table, and wedding cars. It is not only an important form to beautify the wedding scene and add festive atmosphere, but also conveys the future beautiful life yearning and affectionate blessing to a new couple [17, 23]. Meanwhile, In traditional Chinese culture, the ideal realm of life is to obtain the five blessings (Fu in Chinese words): health, wealth, longevity, good morality, and death without disease. In which longevity (Shou in Chinese words) is the core of the five blessings, and the highest realm and ultimate manifestation of the blessing. This forms a traditional auspicious pattern “Five Fu hold Shou” (Five blessings hold longevity) as presented in Fig 8, which is widely spread in Chinese folk. In which the homophonic allegorical expression of traditional Chinese folk art is used. In Chinese, because the word "Bian Fu" for bat is homonym with the word "Fu" for good fortune, and the word “Hu Lu” for gourd has a harmonic similarity to the word “Fu Lu” for blessing, so five bats around the deformed Shou character are used to symbolize the five blessings, while a variant of the Shou character is cleverly incorporated into the bat’s body, and peony flowers are decorated on both sides of their wings. Peony is regarded as a symbol of wealth, representing a rich and beautiful life. The four edge corners are presented in the shape of gourds which are also decorated with Shou character patterns. The whole work cleverly integrates various forms of Shou character elements, in order to emphasize the beautiful meaning of happiness and longevity.

In the funeral rites in Hubei, carved paper-cutting elements are rich and diverse, adding a solemn and mysterious atmosphere to the funeral ceremony. Some common carved paper-cuttings include paper money, paper bundles are mainly used as materialized language to offer sacrifices to ghosts and gods, and to express condolences. Paper money is the most typical representative that symbolizes wealth and blessing. Paper tie is the main material of paper, through cutting, folding, gluing and other methods made into a variety of hades, such as paper tied houses, horses and sheep. Both through burning to provide life needs for the deceased in the underworld [25]. At the same time, some paper-cutting images with the function of exorcising evil spirits may also be used in funeral rituals to protect the dead from evil spirits.

### 3.3 Educational propagenda

As an ancient folk art in China, Hubei carved paper-cutting has been deeply rooted in the traditional cultural concepts of the Chinese nation, playing the educational and civilizing functions with a unique charm that no other ideology can match. The artworks contain not only the external beauty, but also deep rich ideas, ethics, humanistic knowledge and life ideals. This inadvertently influences and shapes people’s world view and values through a unique externalization form, presenting the values in spreading knowledge, inheriting culture and reaching education [26]. In this aspect, the carved paper-cuttings of filial piety theme in Xiaogan City that introduced in Section 2.1 are classics with high reputation.

In the historical period after the founding of PR China, keeping up with the plus of social developments, carved paper-cutting experienced unprecedented prosperity. This promotes a transformation of the folk carved paper-cutting from traditional theme to modern forms. A mass of artworks reflecting the social changes have been created to disseminate and interpretate the new national policies and new ideas, which not only remark the changes of society, but also represent the patriotism and perseverance of the people.

**Fig 9:**
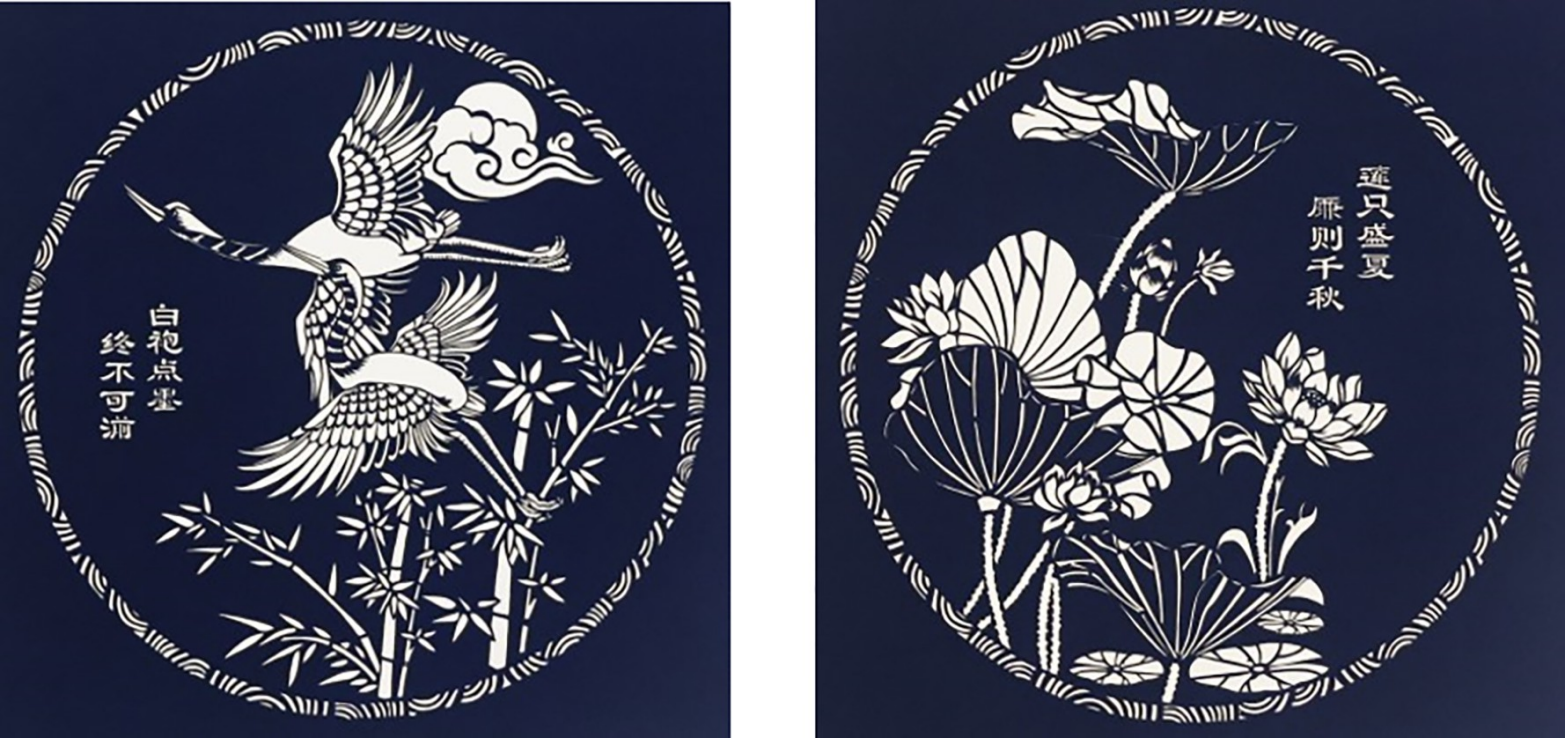
Artworks for QingZhengLianJie education. (a) Crane and Bamboo, (b) Lotus.

With the China coming into new times, the educational roles of carved paper-cuttings are rich and colorful in contents, and quite extensive in subject matter [24]. While telling the Chinese story well, the art of carved paper-cutting becomes a shining pearl with distinct characteristics of educational significance to link between the past and the present, communication between tradition and modernity. Fig 9 shows the artworks saved in Xiantao Mass Art Museum, Hubei, China. These artworks highlight the theme of QingZhengLianJie in the education of government officials. The Chinese word QingZheng means good conduct and integrity, clean and self-disciplined, and impartial; LianJie means to be an innocent man without seeking personal gains and swayed by personal considerations. The crane, bamboo and lotus are all objects representing fair and cleaning in Chinese cultural connotation. The crane is endowed with loyalty, integrity and high moral character. The bamboo is green, tough and upright in a metaphor of people’ integrity and clean. The lotus grows in the mud with white flowers, which are very pure and noble to represent people’ honest on their official career.

## 4. Composition and modelling of Hubei carved paper-cutting

Art does not depart from human creative activity since the birth of human beings, the first tool of human beings has the same identity with the first artwork [27]. The aesthetic function of folk art reflects the aesthetic culture of social groups for a long time. In ancient times, when society was not divided into classes, art was not the exclusive preserve of a specific class, but the joint participation and creation of the whole people. In the long-term practice of production labor, the ancestors mastered the laws of art formal beauty such as rhythm, cadence, symmetry and balance, and produced the sense of art aesthetic form [28]. In Hubei carved paper-cutting, composition and modelling are two important aspects that depend on and complement each other, which together constitute the unique charm and artistic value of the work.

### 4.1 Flexible and diverse composition forms

Composition, as the core element of artistic creation, aims to convey a specific aesthetic and theme through a specific layout and arrangement. In traditional Chinese culture, an aesthetic standard has been always followed that appreciates completeness, symmetry, even number and huge, which originates from the cosmological view revealed by the ancient Taiji philosophy of Yin and Yang. This has been reflected in the previous court art and the creation of literati [29]. Moreover, this simple aesthetic concept is expressed incisively and vividly in the creation of folk art. Originated from the folk of Hubei, carved paper-cutting has both traditional embodiment and flexible innovation in its composition forms.

#### 4.1.1 Center-based composition

Center-based composition is a composition method commonly used in carved paper-cutting. In this kind of composition, a large subject image generally occupies the central position of the picture, which is the focus and key point of expression. Other secondary elements including flowers and plants, vines, auspicious clouds and letter symbols, layout around the subject image. This forms a centripetal composition, as shown in Fig 8. The image is symmetrically arranged for all elements around the central distortion Chinese word “Shou”, which draws attention of observer to the center with fulling of auspicious signs. It can also create an effect of three-dimensional vision to present a vivid picture of happiness.

#### 4.1.2 Symmetric composition

Symmetric composition pursuits a pattern of symmetry and balance, which is one of the most common compositions in Hubei folk art of carved paper-cutting. Symmetry is not only a formal, but also a deep cultural expression. It is usually based on a central point or center line to arrange the pattern appeared left and right or up and down symmetrical shape. Fig 3 presents a symmetric frame with vertical axis of the gourd. Fig 4A) shows the symmetric composition of Children’s saliva pocket with vertical axis. This way of composition gives people a stable, solemn and harmonious feeling, and highlights the pattern theme with a strengthening visual effect.

**Fig 10:**
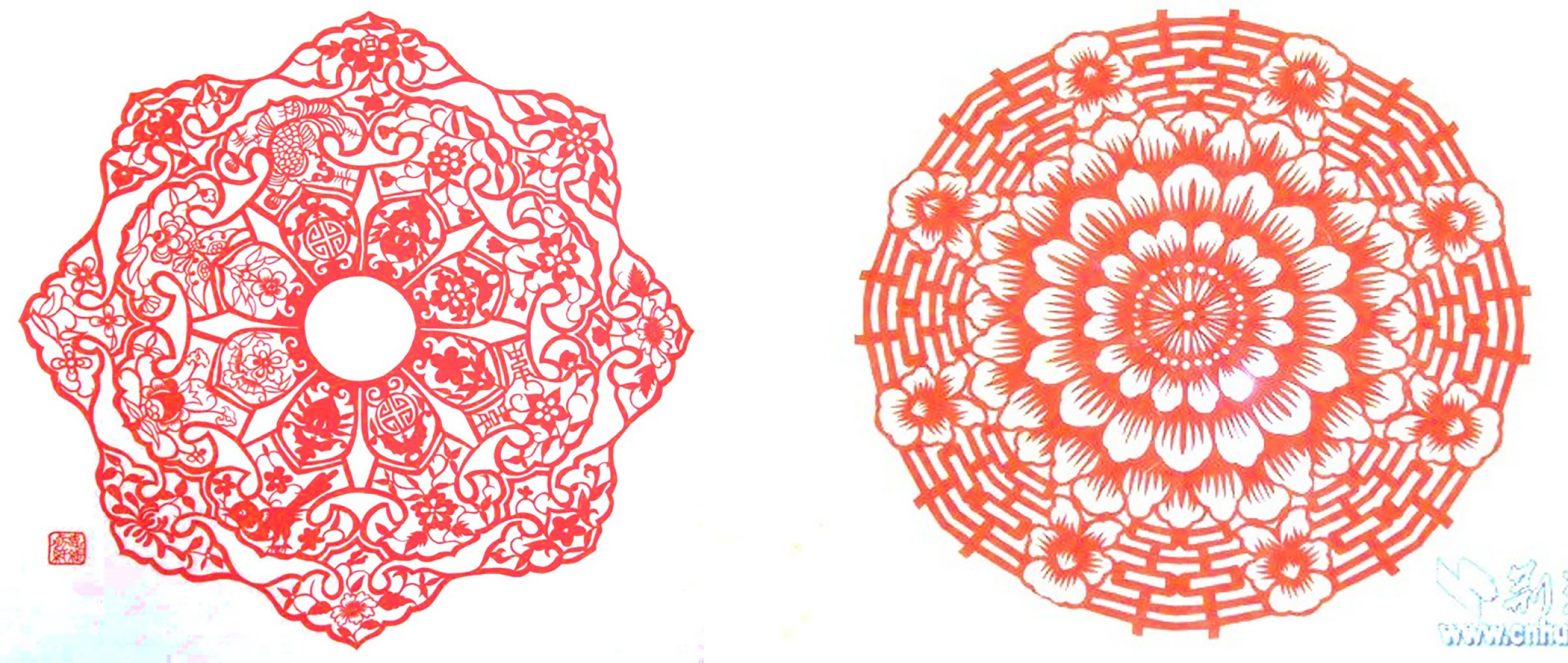
Carved paper-cutting works created by F. Chi and Y. Ma. (**a**) Women’ shawl, (**b**) Flowers.

Fig 10 exhibits the examples of artworks created with a central point to create a radiately symmetric composition, which can be respectively downloaded online: https://www.sohu.com/a/453014705_164838 and https://baijiahao.baidu.com/s?id=1605684607744463572. The artwork of women’ shawl created by F. Chi contains several kinds of meaning patterns including carp, chrysanthemum, peony, longevity words, magpie and copper money. The artwork of flowers created by Y. Ma contains a peony in the middle that surrounded with eight happy flowers and eight Chinese words “Shuangxi” that means very happiness, which is commonly used in wedding celebration to express meanings of auspicious, satisfactory and happiness. Therefore, the symmetric composition perfectly expresses a solemn and harmony image.

#### 4.1.3 Balanced composition

Balanced composition pays attention to the overall coordination and internal balance of pattern, which does not seek the complete symmetry of pattern, but cleverly arrang the position, shape and size of each pattern symbol element, to make the whole pattern in a dynamic visual balance. This can be reflected by Fig 2 that shoes different postures of eight fishes. This way of composition is flexible with strong expressive force. Although the patterns are not equivalent or balanced when viewing them at initial time, the picture presents a dynamic balance with the same quantity of perception, reflecting the formal beauty law of art in change and unity.

#### 4.1.4 Conformal composition

Conformal composition refers to the natural image to abstract out the body skeleton and internal image for creating the pattern of paper-cutting, which shows the respect for the complete and harmonious composition structure. This way of composition subtly uses the physical structure relationship between objects and images, the image can be adjusted with the change of the body itself in a specific morphological space, which is clever to seek changes in the outer frame. They not only retain their own unique characteristics, but also depend on and restrict each other, together constituting a unique form of composition. These elements are reconstructed into a more perfect and profound overall composition, showing the unique charm of paper cutting art. Fig 2 uses the vivid fish shape as the outer frame. Fig 3 uses a gourd forming the outer frame of the map. Fig 4B) uses the square of pillow ends as the boundary. Fig 5 uses the Chinese word Fu as the outer frame. These artworks are all used the conformal compositions to dramatically create the connection of human life with natural environment.

#### 4.1.5 Arbitrary-shape composition

**Fig 11:**
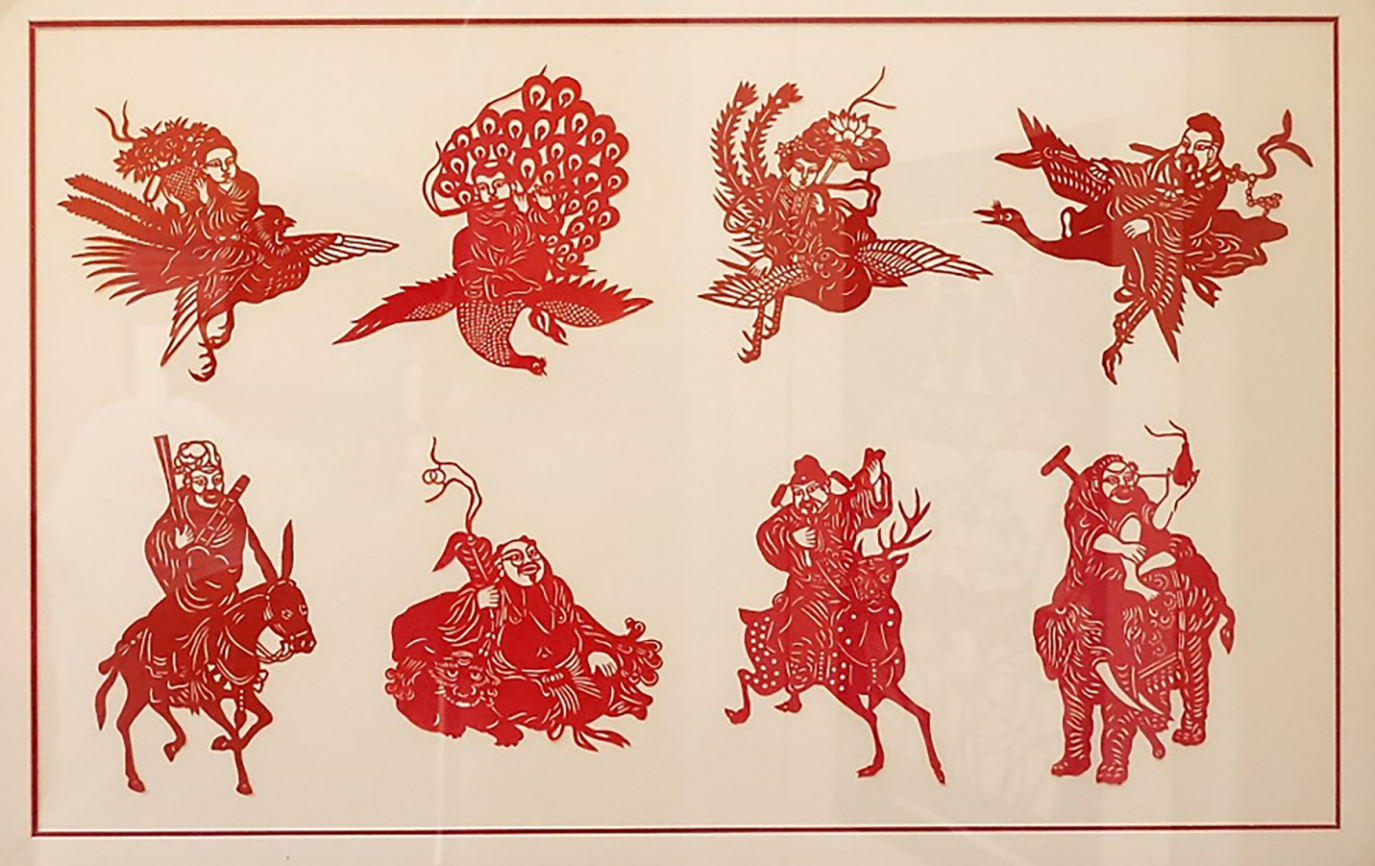
Artwork of " Eight immortals ".

Arbitrary-shape composition has a strong subjectivity, randomness and contingency, resulting in a free and flexible combination of external form and internal structure of the whole picture. It breaks the traditional way of composition to pay more attention to the expression of individuality and freedom. Therefore, it gives people the relaxed and pleasant, lively and clear feelings, and is often used to show objects with dynamic sense and vitality However, arbitrary-shape composition is not random and irregular, it is necessary to create under certain aesthetic and artistic laws to reach the visual harmony and beauty. This requires that creators have better artistic accomplishment and aesthetic ability to transform the natural image into a conformal composition work with artistic appeal, showing the unique artistic style and creativity. The artworks shown in Fig 1 are arbitrary-shape composition with vivid images of characters. The artworks shown in Fig 9 are arbitrary-shape composition with alike form and spirit of crane, bamboo and lotus. Moreover, the artwork of “Eight immortals” saved in Xiantao Mass Art Museum, Xiantao City, China, shown as picture of Fig 11, is also an arbitrary-shape composition which individually forms each character pattern into a separate whole, not bound by form.

### 4.2 Rich and varied modelling technics

#### 4.2.1 Yin or Yang carving

The carved paper-cutting is mainly engraved, shaped by line, formed surface by producing types. The screen of paper-cutting requires that the Yang grains must be connected, and the Yin grains must be broken, and do not scatter when lifting up.

#### 4.2.2 Broken skill

**Fig 12:**
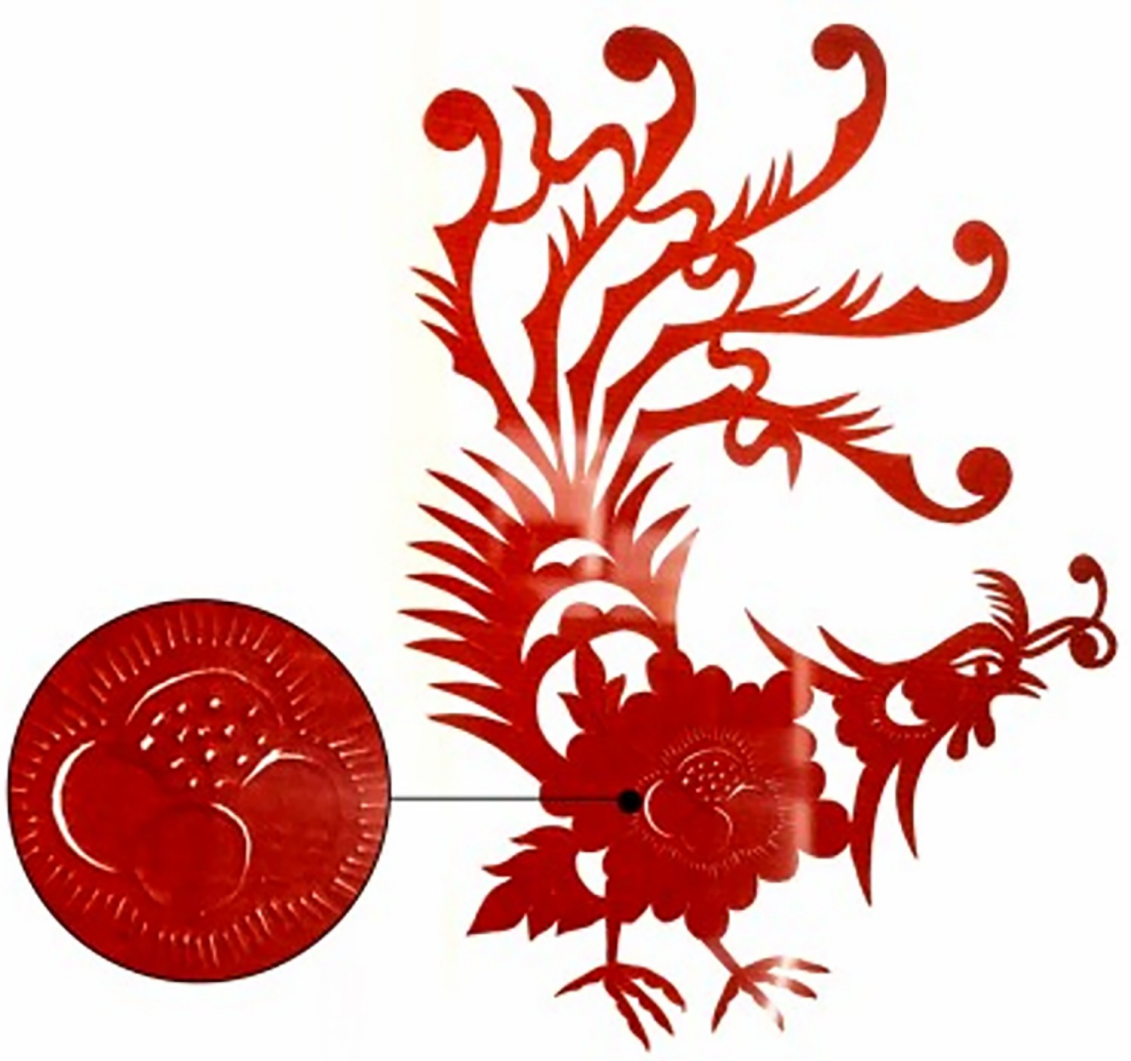
The phoenix.

Broken skill was originally a fine grain specially poked out in order to facilitate the embroidery line. As the Phoenix shown in Fig 12 saved in Xiaogan Carved Paper-cutting Heritage Center, Xiaogan City, China, the left part shows details of the Phoenix body with grain pattern. Later, the broken skill gradually evolved into a unique modelling technic of carved paper-cutting, usually to show stamens, plant textures or feathers, so that the picture is more vivid and delicate.

#### 4.2.3 Unobstructive perspective

**Fig 13:**
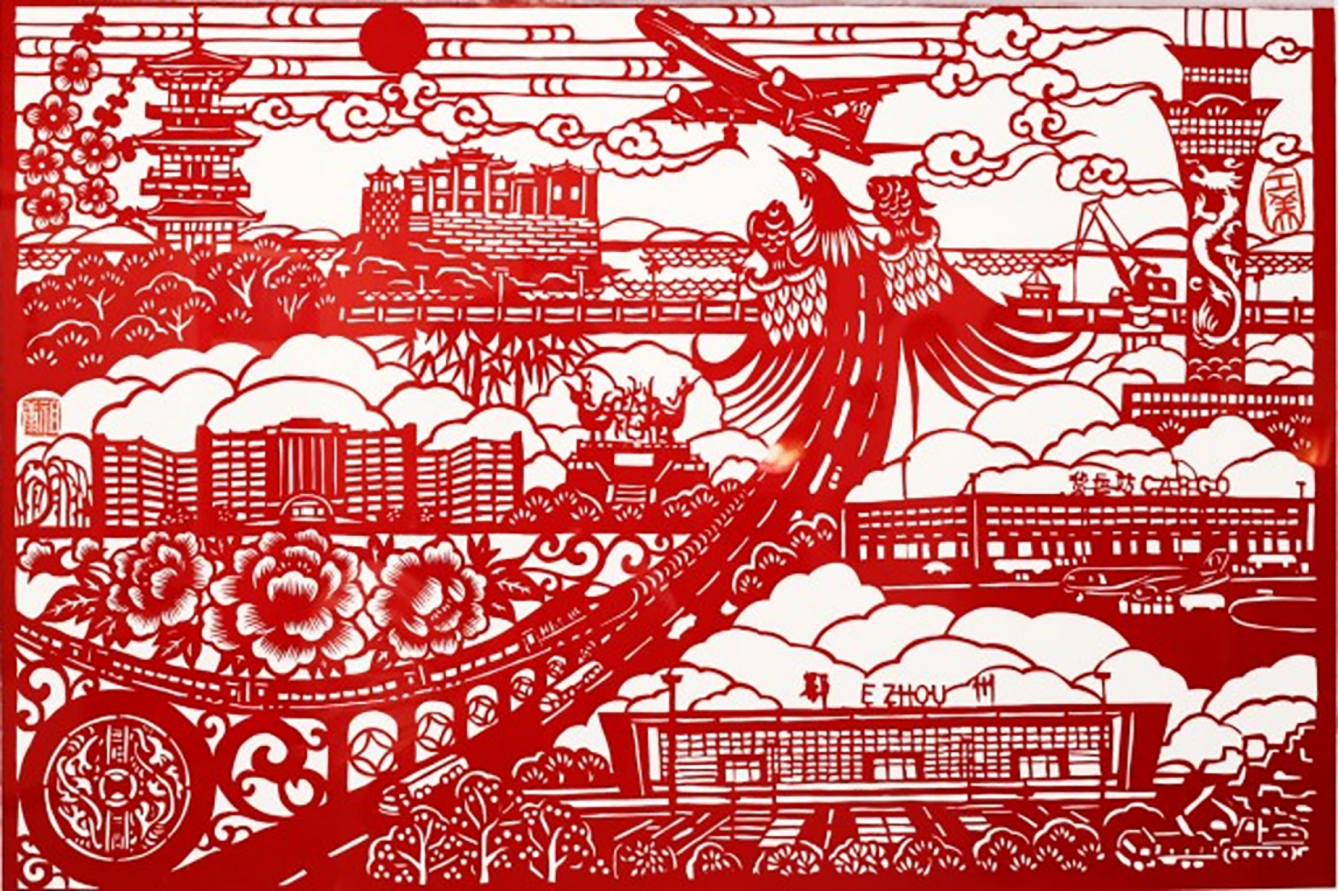
“Soaring Ezhou” created by Z. Xia.

Differed from drawing, carved paper-cutting is difficult to represent the overlapping layers of three-dimensional space, scenes and images. Therefore, in the picture processing, the picture of carved paper-cutting is often presented in a “full view”, which is usually in sharp contrast to the "scientific perspective" followed by classic art in the proportion and perspective relationship between objects and images, and is not subject to the principles, laws or concepts followed by modern painting theory. The scenes of Fig 5 inside the fish frame are carved using unobstructive perspective modelling. Meanwhile, the carved paper-cutting work of Fig 13 saved in E’zhou Huarong Intangible Cultural Heritage Exhibition Hall, E’zhou City, China, the buildings, airplane, phoneix, flowers, tress and plants are all presented in a picture, and do not follow the principle of perspective commonly used in painting. This kind of "narrative language" that can be fully presented and grasped is to express the three-dimensional world with "two-dimensional tiling" visual thinking. That is, to show all kinds of objects on a two-dimensional screen without obstructing each other, and to build a "super time and space" and "surreal" layout style that transcends conventional logical constraints such as size, distance, inside and outside, virtual and real.

This way of modelling contains many technics including non-scale perspective, multi-dimensional perspective, across-time and space perspective, circumferential perspective and light perspective [30]. Each technic is cleverly used and reflected in the art of paper-cutting, displaying the carved paper-cutting creator’s self, spontaneous and instinctive intuitive ideas. The full view of perspective makes the carved paper-cutting show a unique beauty and artistic charm.

#### 4.2.4 Distortion or exaggeration

**Fig 14:**
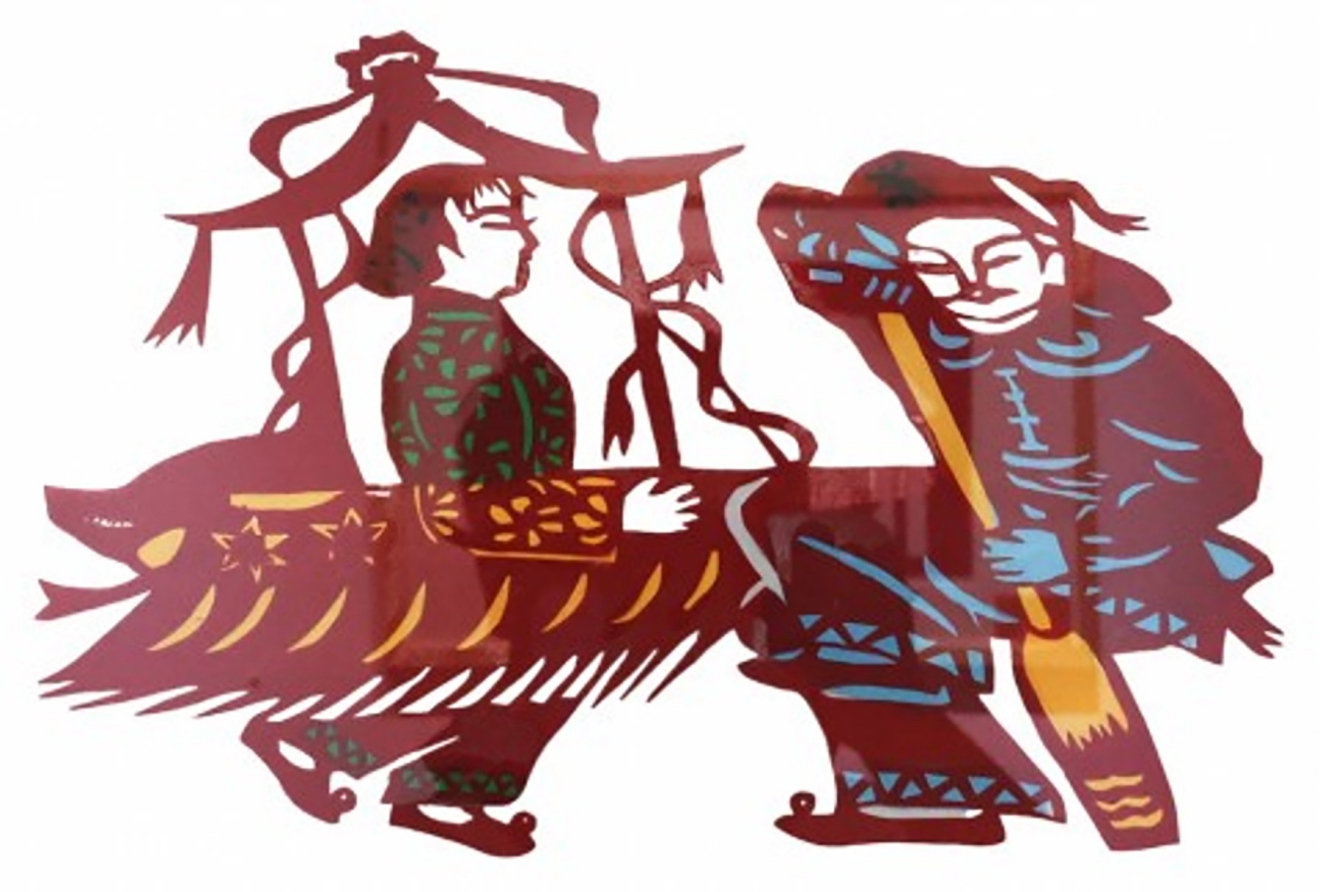
"Rowing a lotus boat, an abundant harvest of grains " created by Z. Duan.

The distortion or exaggeration is also a common modelling method in the art of carved paper-cutting, which uses imagination to exaggerate or shrink the features of objects to highlight their most essential and characteristic parts. This distortion or exaggeration is not only to make the image more vivid and lively, but also a way for the paper-cutting artists to express their feelings, wishes and ideals. Due to the limitations of tools and materials, the exaggeration often captures the main part of the image, and boldly ignores the secondary part to make the subject at a glance. The posture of the image should be exaggerated and beautiful, the movement should be large and full of dynamic sense. As the work of Fig 14 save in Wuhan Mass Art Museum, Wuhan City, China, the exaggeration of characters, boat and grains reserves the features with a high identification followed certain art laws and aesthetic principles, representing the creator’s creative intention and emotional expression. Therefore, the object image is purposefully deformed to achieve a better artistic result.

#### 4.2.5 Association and imagination

**Fig 15:**
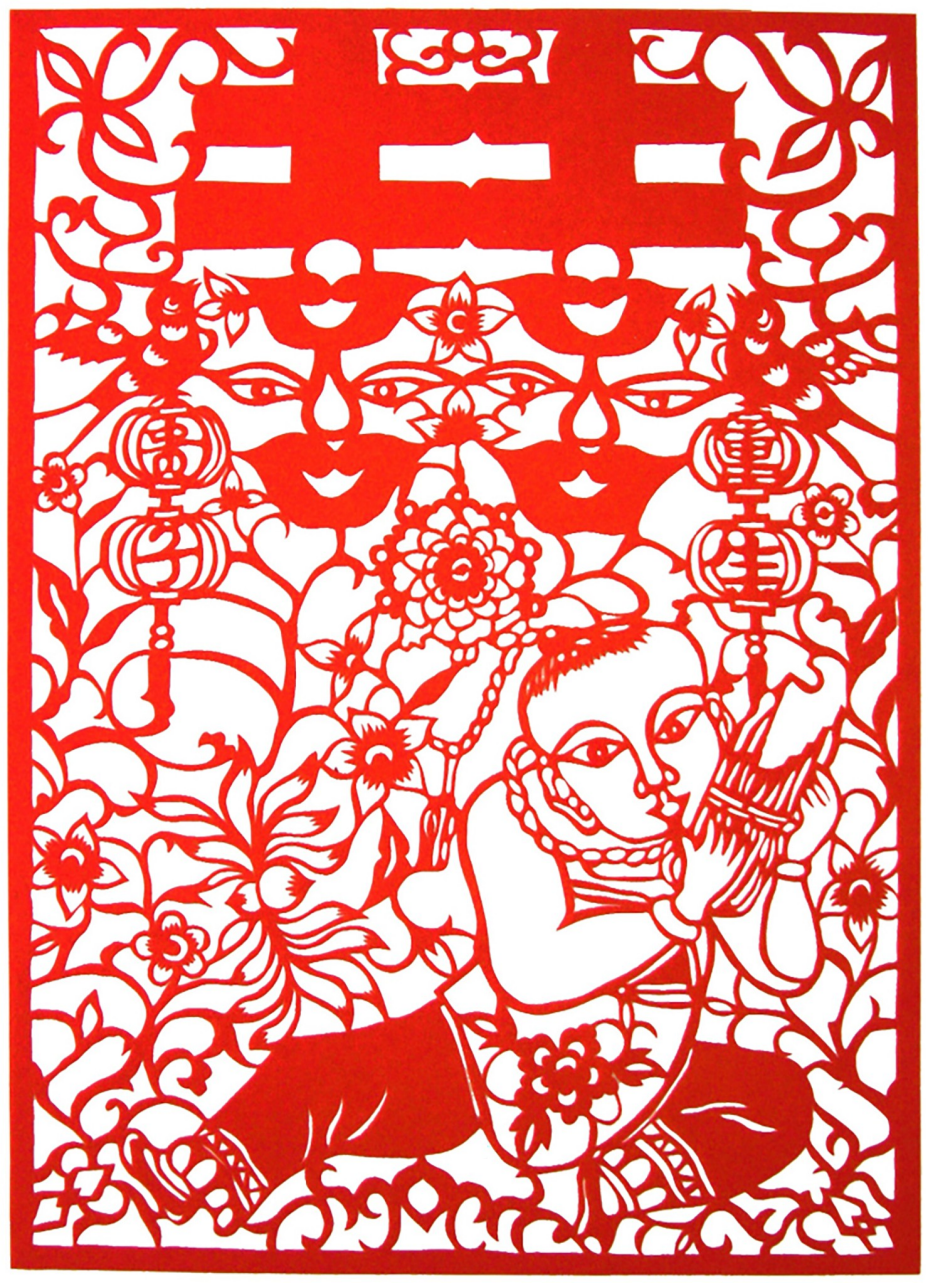
The artwork “happy flower” created by H. Tu and H. Xu.

During the carved process of paper-cutting, the creators do not satisfy to simply reproduce objective reality. In fact, the natural objects are often subjectively transformed to create paper-cutting images with unique personality and artistic appeal through the association and imagination of creators. This originates from the creators’ strong affirmation of their self-subjective consciousness and forms a unique way of thinking and observation, which lays the foundation for the modeling characteristics of "writing soul with will" [29]. Therefore, the external image of realistic and objective things is not the reference object of carved paper-cutting art creation, but the starting point to stimulate the author’s creative association. As the artwork of Fig 15 saved in the Cultural Center of Xiaogan District, Xiaogan City, the Chinese word “Shuangxi" meaning happiness is expressed as hieroglyphic characters of human figures in middle and lit candles in bottom. Accompanied with the hanging lovebirds and lanterns engraved Chinese words meaning "early birth" and "noble son", the upper half part of the picture is constructed using the traditional symmetric composition in vertical axis. In the bottom half part, a little boy wearing traditional belly band and playing sheng music happily adds more liveliness and childlike interest. Meanwhile, flowers and vines are continuously interconnected to spread out a vibrant, harmonious and happy scene. This surreal artistic treatment technique transcends the bondage of natural form, and greatly enhances the visual impact of the work by strengthening the formal beauty and decoration, while further strengthening the theme connotation of conveying wedding blessings and beautiful visions. Therefore, this kind of mental image modelling is the creator’s self-feeling and imagination of natural objects, ultimately boiling the aesthetic standard down to their spiritual satisfaction.

## 5. Conclusions

Taking the artworks crated in Xiaogan, E’zhou and Xiantao cities as representation, the features of Hubei folk art of carved paper-cutting are extracted in this study using an empirical comparative analysis. The results of this study provide a multi-dimensional perspective for the modern inheritance and development of traditional Hubei artworks of carved paper-cutting with specified characteristics.

The regional features of artworks created in these three cities are apparent due to the different folklore culture, labor production and surrounding environment. Although the theme of carved paper-cutting is often the same to pray good lucky and avoid disasters, the carriers are the familiar things to each regional people, resulting in the different expression styles that are much closer to the daily life with specific characters.

The artistic values of carved paper-cutting in the three regions of Hubei are the same in beautification of life, folklore communication and public education, which are generated from the desire of beautiful and happy life. The artworks have high identification using the common symbols that are familiar to Chinese people, including Chinese words, flowers and animals which represent happiness, lucky or evil.

The composition of carved paper-cutting works is flexible and diverse, which is often represented by those of center-based, symmetric, balanced or conformal. They are applied based on the content of works expressed and the shape of carrier.

The modelling technics of carved paper-cutting works are rich and variable in Yin and Yang carving, broken skill, unobstructive perspective, distortion, exaggeration, association, imagination and image. Which are fluent in knife technic, giving an expression in delicate and beautiful, finer in a subtle way, harmony between virtual and real, and neat with complexity, with rich themes and forms and high artistic appreciation value.

However, this work is limited to qualitative research in the feature extraction from a vast of artworks of Hubei carved paper-cutting, which much relies on the authors’ experiences and academic attainments. Therefore, the continued work should explore the possibility of using image processing technics to extract the studied features in automatic way, and enlarge the comparation of similar artworks of paper-cutting in other regions.

## References

[1] Zhang F. Paper-cutting Arts in China and Abroad. Liaoning Art Press: Shenyang, China, 2000.

[2] Yang Y, Bai L. On inheritance and protection of Hubei folk paper-cutting arts. J. Wuhan Univ. Techno. (Social Sci. Ed.) 2013; 26(3): 497–500. 10.3963/j.issn.1671-6477.2013.03.033.

[3] Huang Y, Li G. Carving Gold to Sheng for the dissemination of Jing customs: Research on the inheritance and development of Hubei carved paper-cutting art. Westleather 2020; 42(8): 142–143.

[4] Du Y. Deduction of Jingchu Carved Paper-cut Visual Language in Multidimensional Space. Thesis for Master Degree of Art Design, Wuhan University of Technology, Wuhan, China, 2020.

[5] Zong L (the Nan Dynasty). Translation and annotation to Jingchu Suishi Ji by Tan, L. Hubei People Press: Wuhan, China, 1985.

[6] Liu Y., Study on Folk Art Recorded in Jingchu Suishi Ji. Thesis for Master Degree of Fine Arts, Chongqing Normal University, Chongqing, China, 2018.

[7] Zhou M (the Southern Song Dynasty). Annotation to Old Martial Arts by Fu L. Shangdong Youyi Press: Jinan, China, 2001.

[8] Qi Y. An Analysis of Visual Language and an Extend Design on Carving Paper-cut in Hubei. Thesis for Master Degree of Art Design, Hubei University of Technology, Wuhan, China, 2019.

[9] Zhou Q. The Study of Protection Strategy on Carving Paper-cut in Xiaogan Area. Thesis for Master Degree of Law, South-Central University of Nationality, Wuhan, China, 2016.

[10] Huang B. From Going Out of the Folk to Returning to the Vernacular: Research on the Evolution of Chinese Folk Paper-cut Arts in One Hundred Years. Thesis for Ph.D Degree in Arts, Shanghai University, Shanghai, China, 2018.

[11] Zhang F, Zhang K. Modern Chinese Paper-cutting. Hunan Art Press: Changsha, China, 2003.

[12] Zhou Y, Chen L. Research on the inheritance and development of intangible cultural heritage boosted by science and technology: Taking Xiaogan carved paper-cutting as an example. J. Yangtze Univ. (Social Sci.), 2020; 43(4): 33–37.

[13] Du L. Analysis of filial piety culture connotation of Xiaogan carving paper cut. J. Xiaogan Univ. 2009; 29(5): 36–38.

[14] Li S. On the artistic language of Ezhou folk carved paper-cutting. J. E’zhou Univ. 2012; 19(3): 66–68.

[15] Huang C. Research on excavation, arrangement and digital protection of Mianyang carved paper-cutting. J. Hubei Univ. Nationalities (Ph. Social Sci.) 2015; 33(3): 24–26.

[16] Yang Z, Liu Y. Research on the ideological and political connotation of Hubei carved paper-cut art. Adv. Edu. 2023; 13(10): 7321–7330.

[17] Sun J. Folklore and customs in Xiaogan carving and paper-cut. Decoration 2014; (258): 137–138.

[18] Gao Y. Feature extraction technology-guided visual communication design for folk paper-cutting. Sci. Program. 2022, Article ID 3210054, 9 pages, doi: 10.1155/2022/3210054

[19] Liao Y, Yan L, Hou Z, Shi S, Fu Z, Ma Y. CutGAN: dual-Branch generative adversarial network for paper-cut image generation. Multimedia Tools Appl. 2023, doi: 10.1007/s11042-023-17746-z

[20] Lu S, Lo C, Syu JY, Project-based learning oriented STEAM: the case of micro-bit paper-cutting lamp. Intern. J. Techno. Des. Edu. 2022; (32): 2553–2575. 10.1007/s10798-021-09714-1.

[21] Hu X. Tell Yong Dong’s story, spread the good reputation of Xiaogan. Available online: http://www.xiaogan.gov.cn/llsk/372812.jhtml; 2019-11-15.

[22] Ye X. Brief History of E’zhou Culture. Hubei People Press: Wuhan, China, 2021.

[23] Wang J. Tutorial of Xiantao Paper-cutting. Xiantao Mass Art Museum: Xiantao, Hubei Province, China,2021.

[24] Yang Z, Liu Y. Research on the application of paper-cut illustration in cultural and creative products. Art Res. Letters 2023; 12(4): 308–314.

[25] Wu Z, Li H, Chu Feng Chu Shu/ The Folk of Chu: Large-scale Folk Photography Exhibition; Hubei Art Press: Wuhan, China, 2013.

[26] Wang P. General Argument of Chinese folk Art. China University of Science and Technology Press: Hefei, China, 2007.

[27] Deng F. Art Before Art; Shangdong People Press: Jinan, China, 1986.

[28] Zhong J. Introduction to Folklore; Shanghai Literature and Art Press: Shanghai, China, 2009.

[29] Lv S. See Tradition Again, Part 4; SDX Joint Publishing Company: Beijing, China,2004.

[30] Wang G. Cultural Interpretation of Paper-cutting Folklore. Beijing University Press: Beijing, China, 2009.

